# Authors' Response to Peer Reviews of “Measuring Integrated Novel Dimensions in Neurodevelopmental and Stress-Related Mental Disorders (MIND-SET): Protocol for a Cross-sectional Comorbidity Study From a Research Domain Criteria Perspective”

**DOI:** 10.2196/36212

**Published:** 2022-03-29

**Authors:** Philip van Eijndhoven, Rose Collard, Janna Vrijsen, Dirk E M Geurts, Alejandro Arias Vasquez, Arnt Schellekens, Eva van den Munckhof, Sophie Brolsma, Fleur Duyser, Annemiek Bergman, Jasper van Oort, Indira Tendolkar, Aart Schene

**Affiliations:** 1 Department of Psychiatry Donders Institute for Brain, Cognition and Behaviour Radboud University Nijmegen Netherlands; 2 Pro Persona Mental Health Care Depression Expertise Centre Nijmegen Netherlands; 3 Nijmegen Institute of Scientist-Practitioners in Addiction Radboud University Nijmegen Netherlands; 4 LVR-Klinikum Essen Department of Psychiatry and Psychotherapy University Hospital Essen Essen Germany

**Keywords:** psychiatry, mental health, psychiatric disorders, neuropsychology, stress, comorbidity

*This is the authors' response to peer-review reports for “Measuring Integrated Novel Dimensions in Neurodevelopmental and Stress-Related Mental Disorders (MIND-SET): Protocol for a Cross-sectional Comorbidity Study From a Research Domain Criteria Perspective.”*

## Round 1 Review

### Reviewer B [1]

Building on the Research Domain Criteria (RDoC; Cuthbert and Insel [2]), the manuscript [3] presents the study protocol of a transdiagnostic study program to determine mechanisms that either differentiate between neurodevelopmental and stress-related psychiatric disorders or show commonalities. The authors formulate a compelling argument that the pathophysiological pathway of psychiatric disorder needs to be considered taking a developmental perspective, with an emphasis on the role of comorbidities. To address such a high level of complexity, the authors present a cross-sectional study focused on stress-related (mood, anxiety, and substance abuse) and neurodevelopmental (autism, attention-deficit/hyperactivity disorder) disorders, with four points of measurements (distance unclear), and with each point of measurement including several observational levels: genetics, physiology, neuropsychology, system-level neuroimaging, behavior, self-report, and experimental neurocognitive paradigms.

Reaction: We have now added the time period (1 month) to the procedure section.

Overall, I find this to be an extremely ambitious project. The study protocol as it is provides some good direction, and the approaches taken are state of the art, but the details of the proposal are inaccessible because of its complexity. What worries me most about the ambition of the plan is that the sample size and the requirements of the sample size are not discussed, which leads to issues with the interpretability of the collected data. An issue in a project that puts so much strain on the participants should be carefully considered.

Reaction: We thank the reviewer for the compliments. We must stress that this protocol should be considered as an umbrella for several separate studies and therefore does not permit going into every detail of all envisioned studies. Instead, we have tried to express the general lines of our transdiagnostic approach along the RDoC framework and moreover give enough details about the exact data collection as a reference for other researchers and so that we can refer to this protocol in later papers. Below, we specifically address the sample size issue.

I found the submission to be a mismatch to JMIRx Med; this is clearly a research protocol and might be better suited for JMIR Research Protocols.

Reaction: We would like publish our protocol where it is best suited and will conform to the editor decision here.

Looking at the work solely from a research protocol perspective, I would like to read more details about how the authors intend to combine data or a detailed description of how they intend to pursue their analysis. The complexity prevents them from doing so, but as a result, the quality of the research protocol is difficult to judge—it is too high level to judge all aspects of the protocol responsibly. Defining the most relevant end points would be one approach that would help here.

Either way, I think the work is relevant to address, but journal fit and my mentioned points about sample and approach should be addressed, and the overall work would benefit from formatting and editing (some sections, for example, on the methods used, are redundant).

Reaction: We have restructured the entire manuscript, edited sections, removed redundancy, and moved a section to the discussion. We focus more on statistical analyses that can combine multiple modalities and different levels of observation, such as canonical covariate analysis, linked independent component analysis, and normative modelling.

**Strengths**

- Very important topic
- The authors pose a number of highly relevant questions
- Engaging summary of effects of individual disorders on pathophysiological and shared effect between disorders
- Considering the complexity of this project, the details are well thought through and the approaches described are reasonable. To assess the quality of each approach taken in detail, a range of expertise is required
- The authors pose a number of highly relevant questions
- The authors pose a number of highly relevant questions

**Major Issues**

- The sample size required is huge and one of the bottlenecks of the suggested approach; while the authors seem to have one unit to recruit participants, it is unclear how many participants would take part. The issue I foresee is that, with that many levels of observation, the complexity of comorbidities, and individual differences, the analysis will remain inconclusive. I would like to hear the authors’ thoughts on the sample size and interpretability of the collected data.
  Reaction: We thank the reviewer for the important remark and have now included a full paragraph on this issue:
  “This research protocol will comprise multiple studies to be conducted across multiple years. The majority of studies will estimate effects at the population level by means of parametric t, F or X2 tests where empirical evidence from our and other centers suggests that typical study sizes of ~20-30 subjects per group can be sufficient to detect relevant between group differences, given typical effect sizes across a variety of data modalities. After consulting a biostatistician, we decided that an overall sample size calculation will be of little value. Also power calculations for studies with MRI are difficult and not used routinely, but here is also consensus that groups of ≥20 usually yield sufficient power in MRI-studies to detect moderate differences in regions of interest. Based on these considerations and to have at least 20 subjects per group in the broadly defined comorbid conditions, we aim to include a total of 650 patients and 150 healthy control participants in the time period between 2016 and 2022. In October 2021 we are at 95% of our target. Many research studies that will be conducted under this proposal will be exploratory in nature, where not much prior reference work is available. In these cases we will use expected effect size estimates and ranges thereof generated from testing small samples in pilot studies in order to inform sample size calculations. In these sample size calculations, we expect that for cross-sectional analyses, a power of 80% and an alpha of 0.05 we will be able to detect small differences with respect to clinical variables and questionnaires.”
- The instruments used for data collection (questionnaires, biodata, etc) are all vaguely described (eg, which questionnaires will be used and, if biosamples are collected, what exactly will they be processed for). The data is provided in a later step—it is unclear to me why the same aspect is described twice with different levels of detail.
- Engaging summary of effects of individual disorders on pathophysiological and shared effect between disorders
- Considering the complexity of this project, the details are well thought through and the approaches described are reasonable. To assess the quality of each approach taken in detail, a range of expertise is required.
- Reaction: We have included a supplemental text with a full description of all the data that is collected and how it will be processed. Throughout the manuscript, we only mention the instruments briefly to avoid redundancy.
- Throughout the paper, it is not clear if the work has been performed, will be performed, or is still in the process of development and approval. This might be partially due to changes in time but also due to the overall presentation of the protocol—being more upfront about the goals of the manuscript would have helped.
  Response: We have ethical approval and aim to include a total of 650 patients and 150 healthy control participants in the time period between 2016 and 2022. In October 2021, we are at 95% of our target. We explicitly state this in the Methods section now.

**Minor Issues**

- The formatting in the Word document and the PDF makes the document difficult to read. The Word document shows incorrect breaks and paragraphs, while the font in the PDF is pixelized.
  Reaction: The formatting of the manuscript was unwantedly changed somewhere during the submission process, and we hope that it is now fixed.
- The citation format is not in line with JMIR standards.
  Reaction: We have adapted the citation format to be in line with JMIR standards.
- Acronyms like RDoC or MIND are not introduced at their first occurrence, which makes the interpretation difficult.
  Reaction: We have gone through the whole manuscript to make sure that all acronyms or abbreviations are properly introduced.
- Classifying autism as a disorder misses a neurodivergent perspective, which the autism community perceives, see [4].
  Reaction: We acknowledge that classifying autism as a disorder misses a neurodivergent perspective, which is of course well in line with our transdiagnostic approach. We now mention this issue in the discussion, using this reference. We also refer to the control group now as neurotypical, which is also in line with comments of the second reviewer, to better accommodate nuances in classifying autism.
  Although autism spectrum disorder (ASD) is primarily characterized by alterations in sensory sensitivity, inflexible routines, restricted interests, and deficits in social functioning or rather neurodivergent social functioning, about 50% of high-functioning adults diagnosed with ASD who were referred to a psychiatry department had comorbid major depressive disorder.

## Reviewer AS [5]

### General Comments

This paper is interesting and sets the stage for a pretty comprehensive study.

### Specific Comments

#### Major Comments

1. The background is very long, and some spaces are redundant, talking about the overlap of symptoms in comorbidities. Some of this may be better in a discussion—there is a lot of information here. Reaction: We thank the reviewer for the feedback and agree that the background is too information dense. We have shortened the background, have removed redundant parts, and have moved some parts to the discussion when these parts mainly concern considerations based on the content overlapping and distinctions in mechanisms between neurodevelopmental and stress-related disorders.
2. There are a lot of definitive/overly positive statements (eg, “...the RDoC frameworks fits ideally...” “...we can disentangle.” Consider rewording as this is a fairly small sample size in a singular area of the world. Reaction: We have reworded too definitive or overly positive statements throughout the manuscript.
3. Adjust the title so it is clear that this is a description of methods. Reaction: We have adjusted the title to indicate that this paper contains a rationale and description of methods: “Measuring Integrated Novel Dimensions in Neurodevelopmental and Stress-related Mental Disorders (MIND-Set): protocol for a cross-sectional comorbidity study from an RDoC perspective”
4. Anticipated limitations should be included (eg, single-center, nondiverse population, or the number of data points making differentiation challenging). Reaction: We have added a limitations section in the discussion that reads as follows: Limitations: This study has to been understood in the light of some limitations. Although we aim for a fairly large sample size (we aim to include a total of 650 patients and 150 neurotypical control participants), specific cells of comorbidity between disorders may be low for group comparisons. Moreover, the participants are all recruited at one psychiatric center, i.e. the psychiatric department of the Radboud university medical center, which specializes in the diagnosis and treatment of neurodevelopmental disorders and stress-related disorders in adults and their comorbidity, and this constitutes a form of selection bias and decrease generalizability of the study results to other populations.

#### Minor Comments

1. Change addiction disorder to substance use disorder. Reaction: We have changed addiction to substance use throughout the manuscript.
2. Provide a citation for the first line about the acceptance of psychiatric comorbidities as common. Reaction: We have provided a reference for the common comorbidity of psychiatric disorders.
3. Define abbreviations upon first use (eg, DSM-5 [Diagnostic and Statistical Manual of Mental Disorders (Fifth Edition)]). Reaction: We have gone through the whole manuscript to make sure that all acronyms or abbreviations are properly introduced.
4. Consider changing “healthy” to “neurotypical.” Personality traits were examined too, but there is not a lot of rationale here regarding overlap. I agree it is important to review this too, but this needs to be discussed. Reaction: We agree that neurotypical is better to describe our control group and have changed this throughout the manuscript. This also fits with the point raised by the other reviewer pointing to neurodiversity when considering autism. The inclusion of both personality traits provides the opportunity to analyze distinct and shared variance with, for example, autistic traits, but also with negative effects. For example, factor analyses may reveal overlapping dimensions here. We have explicitly mentioned this in the Methods section under measure and statistical analysis.
5. Is microbiome included at the very end as a data point? Reaction: We have moved the paragraph on the microbiome to the appropriate section in the manuscript.

## Review Round 2

### Reviewer B

I want to thank the authors for such an in-depth, detailed, and carefully presented protocol. This is such a challenging study, but the presented implementation connects the different levels of inquiry and the patient groups very well. I found the decision made to be well motivated and am satisfied with the improvements.

I have one point that requires clarification:

– The authors aim to work with people diagnosed with ASD but also included the command of language as an exclusion criterion (ie, “inadequate command of the Dutch language”). How will the authors make sure that not only vocal patients with ASD are included? From my understanding, selective mutism is quite common in people with ASD.

Reaction: Indeed, the reviewer is right that the nature of our approach, with several questionnaires, behavioral assessments, and neuropsychological assessments, requires normal intellectual abilities and excludes mutism in people with autism, and it is therefore right to mention this, as we only include patients with high-functioning autism.

We have mentioned this in the Methods section as follows:

“With regard to autism spectrum disorders, our exclusion criteria implicate that we only investigate patients with high functioning autism, without intellectual disability and without mutism.”

Several minor comments: overall, the manuscript requires proofreading and finishing touches.

#### Abstract

“on the basis of” to “based on”

#### Introduction

“the exception (1) .” to “(1).”

“on the basis of” to “based on”

#### Current Approaches

“especially in light of” to “considering”

“Are depressive symptoms in someone with an autism spectrum disorder comparable to depressive symptoms in someone without an autism spectrum disorder?”; I assume that this should be “attention-deficit/hyperactivity disorder” in one of the cases.

“How well is someone with an autism spectrum disorder actually able to recognize and verbalize their mood symptoms, and how does this impact the diagnostic procedure, and the treatment choice and course?”; I suggest removing “actually”—it is unclear what the “actually” emphasizes, that there is little knowledge from a medical standpoint or if it emphasizes the assumption that people with autism are not aware of their own mood. I lack specialization in working with people with autism, but I would suggest to carefully frame neurotypical assumptions about neuroatypical processes.

#### Comorbidity Within the RDoC Framework

“from a genetic, molecular or cellular level” to “from a genetic, molecular, or cellular level”

I stop commenting on this, but the use of the Oxford comma would help with readability when lists are used.

#### Data-Driven Approaches

“has to be understood as step in” to “as a step towards”

#### Study Aims and Outline

“mood, anxiety and substance abuse” to “mood, anxiety, and substance abuse”

#### Methods

“are as well paid a small fee” — is there a reason the exact amount is omitted?

#### Session 2: Behavioral Assessment

“faeces” to “feces”

“the Autism Spectrum Quotient ( AQ-50)” to “(AQ-50)”; “( NIDA)” to “(NIDA)”

“of the negative valence system”; unclear why underlined, maybe a subheading would differentiate the different systems discussed here better

#### General Issues

Use of Oxford comma in lists

eg and ie should be followed by a comma. See [6].

Check the document for double spaces.

Reaction: We thank the reviewer for the careful reading of the manuscript and the suggestions. We have gone through the manuscript and have adapted all the mentioned issues by the reviewer.

## Abbreviations

ASD: autism spectrum disorder
DSM-5: Diagnostic and Statistical Manual of Mental Disorders (Fifth Edition)
RDoC: Research Domain Criteria
